# Coping flexibility and associated factors after gastrectomy in patients with gastric cancer: A cross-sectional multisite study

**DOI:** 10.1016/j.apjon.2024.100627

**Published:** 2024-11-29

**Authors:** Miwako Eto, Sena Yamamoto, Ryohei Kawabata, Tamon Miyanaga, Noriko Iga, Aoi Yoshino, Hiroshi Yamada, Yoko Nishitani, Mami Matsunaga, Harue Arao

**Affiliations:** a Division of Health Sciences, Osaka University Graduate School of Medicine, Suita, Osaka, Japan; b Nursing Department, Bell-land General Hospital, Sakai, Osaka, Japan; c Department of Surgery, Sakai City Medical Center, Sakai, Osaka, Japan; d Division of Surgery and Cancer Care Center, Fukui Prefectural Hospital, Fukui, Japan; e Nursing Department, Kishiwada City Hospital, Kishiwada, Osaka, Japan; f Nursing Department, Ikeda City Hospital, Ikeda, Osaka, Japan; g Nursing Department, Kita-Harima Medical Center, Ono, Hyogo, Japan; h Nursing Department, Japanese Red Cross Kyoto Daini Hospital, Kyoto, Japan; i Nursing Department, Fuchu Hospital, Izumi, Osaka, Japan

**Keywords:** Coping flexibility, Postgastrectomy dysfunction, Health literacy, Patient education

## Abstract

**Objective:**

To elucidate the current state of coping flexibility and associated factors in gastric cancer patients after gastrectomy.

**Methods:**

A cross-sectional multisite study was conducted with 142 patients with gastric cancer who completed questionnaires on coping flexibility, postgastrectomy dysfunction, health literacy, and perceived social support. Coping flexibility was measured using the Coping Flexibility Scale-Revised, which includes three subscales: Abandonment Coping (i.e., abandoning ineffective coping strategies), Re-Coping (i.e., assessing the process of developing and implementing alternative coping strategies), and Meta-Coping (i.e., monitoring coping flexibility process). Higher scores indicate greater coping flexibility. Descriptive statistics and univariable and multivariable logistic analyses were conducted to examine factors associated with coping flexibility.

**Results:**

The mean age of the participants was 72.6 (± 10.5) years. Limited activity due to decreased food consumption was associated with lower scores for Abandonment Coping (odds ratio [OR]: 0.4; *P* ​= ​0.03; 95% confidence interval [CI], 0.2–0.9) and Re-Coping (OR: 0.3; *P* ​= ​0.003; 95% CI, 0.1–0.6). Higher levels of communicative health literacy were associated with higher Abandonment Coping scores (OR: 1.1; *P* ​= ​0.04; 95% CI, 1.0–1.3), and higher levels of critical health literacy were associated with higher Re-Coping scores (OR: 1.2; *P* ​= ​0.03; 95% CI, 1.0–1.3).

**Conclusions:**

Limited activity due to decreased food consumption and limited coping flexibility were significantly associated. Health literacy had an important role in facilitating coping flexibility. Nursing support may be crucial in evaluating coping strategies and developing alternatives based on new information.

## Introduction

Cancer is the leading cause of death worldwide.^1^ Among the various cancer types, gastric cancer has shown significant increases in incidence and mortality rates. The number of gastric cancer cases increased from 880,000 in 1990 to 1.3 million in 2019, and the number of deaths caused by this disease has increased from 788,317 to 957,185 during the same period.^2^ Gastric cancer is the third leading cause of cancer-related deaths worldwide.^3^ East Asia had the highest incidence of gastric cancer in 2019, with 626,489 cases.^2^ The top three countries for gastric cancer incidence in 2019 were China, India, and Japan, which together accounted for 61.5% of global cases and 58.6% of global deaths.^2^ While mortality rates from gastric cancer are decreasing in Asian regions due to early detection and improved treatment, the overall incidence rates remain high. In Japan, the incidence rate among people aged ≥ 80 years is increasing, indicating that gastric cancer remains a major health problem.^2^^,^^3^

The standard treatment for stage I–III gastric cancer is primarily gastrectomy, with adjuvant chemotherapy recommended for stage II and higher to prevent recurrence.^3^^,^^4^ After gastrectomy, patients often experience complications, such as dumping syndrome, reflux symptoms, and small stomach syndrome, due to the removal of the extrinsic and intrinsic nerves and gastrointestinal hormone-secreting areas.^5^, ^6^, ^7^ These complications can limit oral intake and nutrient absorption, thereby resulting in malnutrition. Patients attempt to consume food to aid recovery; however, permanent changes in stomach function and fluctuating digestive and bowel problems make adjusting their diet appropriately challenging. Eating can cause pain and dysphagia so that, rather than meeting a person's physiological needs, it becomes a source of distress.^8^ Malnutrition can decrease physical and mental strength, which can result in a reduced range of activities, inability to fulfil social roles, and psychological distress. As weight loss progresses, changes in physical appearance can have a further negative impact on a patients' quality of life. The prevalence of depression and anxiety among patients with gastric cancer is particularly high, with approximately 40% and 30% experiencing depression and anxiety, respectively, indicating considerable difficulties with psychological adaptation.^9^^,^^10^ This stressful situation can complicate treatment continuation and ultimately affect prognosis. Therefore, nursing support after gastrectomy is crucial in helping patients with gastric cancer develop coping strategies for fluctuating physical symptoms and facilitate adaptation to life after surgery.

Coping flexibility has a crucial role in managing persistent stress and rebuilding life after gastrectomy. The concept of coping flexibility refers to the ability to select appropriate coping strategies, based on the evaluation of stressful situations, and to balance them well.^11^, ^12^, ^13^ Research on coping with stress has examined the nature and extent of physically and mentally challenging environmental stimuli (i.e., stressors), perceived and assessed difficulty in coping with them, coping strategies, and outcomes of stress reactions. However, research on coping flexibility has emphasised the diversity and fluidity of coping mechanisms in stressful situations, including chronic distress, post-disaster posttraumatic stress disorder, and grief after bereavement.^14^, ^15^, ^16^ Studies focusing on patients with cancer have shown that individual resources are associated with greater flexibility^17^ and that greater coping flexibility is correlated with less variability in physical symptoms.^18^ Previous research has suggested that the ability to recognise situational changes, appropriately evaluate coping effectiveness, and use situation-specific coping strategies to avoid fixation on a single strategy for specific stressors leads to psychological adaptation.^12^^,^^13^^,^^19^^,^^20^ Kato's dual-process theory is supported by research on chronic diseases.^15^^,^^21^^,^^22^ The results of these studies suggested that the concept of coping flexibility may apply to nursing care and research with cancer patients. However, the coping flexibility of gastric cancer patients experiencing a permanent change in physical function of gastrectomy is not fully understood. Investigating and clarifying coping flexibility and related factors in patients who have undergone gastrectomy in Japan, a country with a high incidence of gastric cancer, may provide valuable insights into managing distress and promoting psychological adjustment in these patients. These findings suggest that the concept of coping flexibility can be applied to nursing care for postgastrectomy patients to assist in their distress management and psychological adaptation.

Coping flexibility has been associated with various factors, including age,^15^^,^^23^ sex,^24^ and social support.^24^ Research suggests that coping strategies vary between individuals and groups and are influenced by the nature of stressors and cultural factors.^24^ In addition, health literacy is thought to be related to coping flexibility as patients who can obtain information from health care providers, evaluate its appropriateness, and incorporate it into their coping strategies may be able to expand their coping repertoire.^25^^,^^26^ Coping flexibility may be related to health literacy, which is the ability to access, understand, and use health-related information as a basis for implementing effective coping strategies. Specifically, the ability to access, understand, and use information relevant to postgastrectomy management may enhance patients’ capacity to evaluate the appropriateness of coping strategies in their personal context and implement appropriate coping strategies.^26^, ^27^, ^28^

Improved coping flexibility may enable individuals to abandon ineffective coping strategies, develop and implement appropriate alternative strategies, based on the situation, and regularly review and reflect on coping processes, goals, strategies, and outcomes. This enhanced ability may lead to more appropriate coping strategies for fluctuating situations, potentially facilitating the discovery of trade-offs in postgastrectomy life and aiding adaptation to postoperative changes.

The aim of this study was to elucidate the current state of coping flexibility and its associated factors in cancer patients after gastrectomy. Understanding the current state of coping flexibility and its associated factors in gastric cancer patients after gastrectomy provides insight into the development of tailored interventions to improve patients' ability to cope with changing circumstances and rebuild their postoperative lives. This research has the potential to provide such insights for developing tailored interventions for patients with gastric cancer in their postoperative lives, and ultimately to enhance patients’ ability to cope with changing situations and rebuild their postoperative lives.

## Methods

### Participants and recruitment

Researchers at eight designated cancer care hospitals used convenience sampling to select potential study participants, based on predefined inclusion and exclusion criteria, between August 2022 and April 2024. The inclusion criteria were as follows: (1) age ≥ 20 years with consent to participate, (2) outpatient diagnosed with stage I–III gastric cancer who underwent gastrectomy, and (3) the questionnaire was completed 3 months to 1 year after their surgery. Patients who underwent adjuvant therapy were included. The exclusion criteria were (1) a diagnosis of postoperative cancer recurrence or progression and (2) the presence of other serious diseases or severe psychiatric symptoms (e.g., severe cognitive impairment, severe depression).

The researchers checked the inclusion and exclusion criteria for the study via medical records and information from the physicians. If the criteria were met, permission to participate in the study was obtained from the attending physicians of eligible candidates, and the purpose of the study was explained to the eligible candidates in written and oral forms. Patients who provided consent to participate in the study were considered eligible. The researchers at each institution explained the study to potential participants during outpatient visits and asked them to complete and return the questionnaire.

The sample size was calculated using G∗Power 3.1.9.7 (University of Dusseldorf, Dusseldorf, Germany),^29^ with an effect size of 0.15, a significance level of 0.05, and a power of 0.8, resulting in a required sample size of 143 participants. The design, conduct, and reporting of this observational study adhered to the Strengthening the Reporting of Observational Studies in Epidemiology (STROBE) guidelines to ensure the transparency and rigour of the research.^30^

### Measures

#### Demographic and disease-related data

Demographic data (e.g., age, sex, marital status, cohabitation status, educational background, employment status, and economic situation) and disease-related information (e.g., percentage of weight change from the preoperative period, postoperative body mass index [BMI], surgical procedure, time since surgery, postoperative adjuvant chemotherapy, and comorbidities) were collected using self-administered questionnaires.

#### Coping flexibility

The Coping Flexibility Scale-Revised (CFSR) was used. The CFSR consists of 12 items, divided into three subscales: Abandonment Coping (4 items assessing the process of abandoning ineffective coping strategies), Re-Coping (4 items assessing the process of developing and implementing alternative coping strategies), and Meta-Coping (4 items assessing the process of monitoring the effectiveness of coping flexibility). Response scores ranged from 0 (‘not applicable’) to 3 (‘very applicable’), with higher scores indicating greater coping flexibility. The subscales had Cronbach's alpha coefficients ranging from 0.86 to 0.92.^11^^,^^13^

#### Postgastrectomy dysfunction

The Dysfunction After Upper Gastrointestinal Surgery 20 (DAUGS20) scoring system was used. This 20-item instrument assesses postgastrectomy dysfunction, based on seven subscales using a 6-point interval scale. Total scores ranged from 0 to 100, with higher scores indicating greater dysfunction. The overall Cronbach's alpha was 0.90 with subscale alpha values ranging from 0.61 to 0.86 for gastroesophageal reflux, limited activity due to decreased food consumption, deglutition dysfunction, symptoms of dumping syndrome, transfer dysfunction, hypoglycaemic symptoms, and diarrhoea symptoms.^31^, ^32^, ^33^, ^34^, ^35^

#### Health literacy

The Functional Communicative and Critical Health Literacy (FCCHL) scale was used. This instrument measures functional (5 items), communicative health literacy (5 items), and critical health literacy (four items) on a four-point scale ranging from 1 (‘never’) to 4 (‘often’). Mean scores were calculated for the total scale and the subscales, with higher scores indicating higher health literacy. The overall Cronbach's alpha was 0.78, with subscale alpha values of 0.84 for functional, 0.77 for communicative, and 0.65 for critical health literacy.^36^

#### Social support

The Japanese version of the Multidimensional Scale of Perceived Social Support (MSPSS) was used. This 12-item scale assesses perceived social support from family, significant others, and friends using a 7-point Likert scale ranging from 1 (‘very strongly disagree’) to 7 (‘very strongly agree’). Higher scores indicate greater perceived social support. The overall Cronbach's alpha was 0.91, with subscale alpha values of 0.94 for family support, 0.88 for support by a significant other, and 0.90 for friend support.^37^

### Data analysis

All data analyses were conducted using SPSS Statistics, version 29 (IBM, Armonk, NY, USA). Descriptive statistics were used for all variables. For the DAUGS20, mean scores were calculated by dividing the subscale score by the number of items in this subscale to facilitate comparisons between subscales. Frequencies and percentages were computed for categorical variables, and the mean and standard deviation were computed for continuous variables. For the CFSR, the distributions of responses for each subscale were determined and categorised as ‘very applicable/applicable’ (scores: 3/2) and ‘slightly applicable/not applicable’ (scores: 1/0).

Univariable and multivariable analyses were conducted to examine the factors associated with coping flexibility. To examine the characteristics and associated factors of each group and develop practical support methods, the group with a higher-than-average score on the CFSR was designated High, and the group with a lower than average score was designated Low.

With this as the outcome variable, univariate logistic regression analysis was conducted using the DAUGS, FCCHL, MSPSS, participants' demographics, and disease-related data as the explanatory variables. All variables with a significance level of *P* ​< ​0.2 in the univariable analysis were included as the explanatory variables in the multiple logistic regression analysis. Before conducting multiple logistic regression analysis, the correlation coefficients between variables were calculated to check for multicollinearity. Before conducting the multivariate logistic regression analysis, correlation coefficients between variables were calculated to check for multicollinearity. Cook's distance, leverage ratio, and DFBeta were computed to ensure that no extreme outliers were exerting undue influence on the data used in the model. The logit of the outcome variable was verified to increase as values of the continuous explanatory variables increased. Patients with missing data were excluded from the analysis. The significance level for all statistical tests was set at 0.05.

### Ethical considerations

The study was conducted in accordance with the principles stated in the Declaration of Helsinki and was approved by the Clinical Research Ethics Committees of several institutions: Bell-land General Hospital (Osaka, Japan; IRB No. 2022–010), Fukui Prefectural Hospital (Fukui, Japan; IRB No. 23–24), Kita-Harima Medical Center (Hyogo, Japan; IRB No. 05–27), Japanese Red Cross Kyoto Daini Hospital (Kyoto, Japan; IRB No. S2022-39), Ikeda City Hospital (Osaka, Japan; IRB No. 3462), Sakai City Medical Center (Osaka, Japan; IRB No. 22–322), and Kishiwada City Hospital (Osaka, Japan; IRB No. 36). Fuchu Hospital (Osaka, Japan) approved the study, based on the results of an ethical review by Bell-land General Hospital.

All potential participants were informed, verbally and in writing, of the purpose and procedures of the study during their outpatient visits. They were assured that their participation was voluntary and that refusal to participate or withdrawal from the study would not affect their medical care. All participants were assured of their anonymity. Informed consent was obtained through the completion of a consent form integrated into the questionnaire.

## Results

**Fig. 1:**
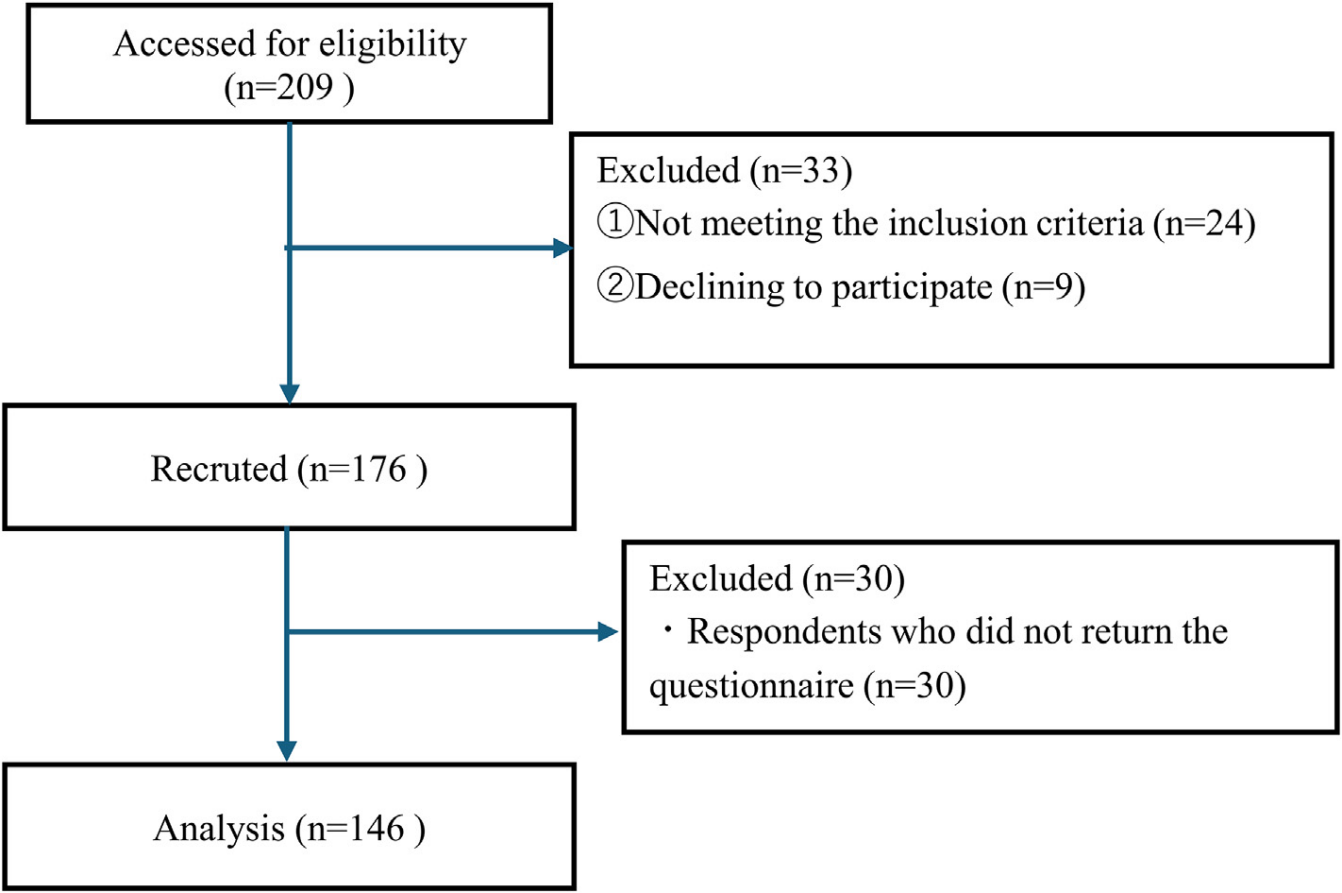
Study flow diagram.

Of the 176 questionnaires distributed to eligible candidates, 142 questionnaires were returned (response rate: 80.7%) (Fig. 1).

### Demographic and clinical characteristics

Table 1 summarises the demographic and clinical characteristics of the participants. The mean age ± standard deviation was 72.6 ​± ​10.5 years. Most participants were men (71.1%), married (73.2%), living with others (81.0%), and had at least a high school education (74.7%). Most (82.4%) participants underwent laparoscopic or robotic surgery for procedures other than total gastrectomy (77.5%). The mean weight change rate from the pre- to postoperative period was −10.9% ​± ​9.2%, and the mean postoperative BMI was 20.2 ​± ​2.7 kg/m^2^.

**Table 1:** Sociodemographic and clinical characteristics of the participants (*N* ​= ​142).

Variable		Mean (SD) or *n* (%)
Age, years (*n*=138)		72.6 (10.5)
		(Range, 32–90)
Sex	Male	101 (71.1)
	Female	41 (28.9)
Marital status	Married	104 (73.2)
	Not married	35 (24.6)
	Missing values	3 (2.1)
Cohabitant	Lives with a cohabitant	115 (81.0)
	No cohabitant	24 (16.9)
	Missing values	3 (2.1)
Occupational status	Currently employed	40 (28.2)
	No current employment	87 (61.3)
	Other	13 (9.2)
	Missing values	2 (1.4)
Annual household income, yen	< 1,000,0001,000,000–1,990,000	16 (11.3)33 (23.2)
	2,000,000–3,990,000	56 (39.4)
	4,000,000–5,990,000	17 (12.0)
	6,000,000–7,990,000	7 (4.9)
	≥ 8,000,000	4 (2.8)
	Missing values	9 (6.3)
Education level	Primary school/junior high school	32 (22.5)
	High school	67 (47.2)
	College	14 (9.9)
	Bachelor/Master	25 (17.6)
	Other	2 (1.4)
	Missing values	2 (1.4)
Total gastrectomy	Yes	28 (19.7)
	No	110 (77.5)
	Missing values	4 (2.8)
Laparoscopic or robotic surgery	YesNo	117 (82.4)20 (14.1)
	Missing values	5 (3.5)
Rate of weight change,		−10.9 (9.2)
% (*n*=138)		(Range, ​−33.3 to ​−38.8)
Postoperative BMI,		20.2 (2.7)
kg/m^2^ (*n*=138)		(Range, 14.0–28.6)
Current anticancer treatment	Undergoing treatment	48 (33.8)
	No treatment	78 (54.9)
	Other	11 (7.7)
	Missing values	5 (3.5)
Comorbidity	Presence of comorbidities	96 (67.6)
	No comorbidities	42 (29.6)
	Missing values	4 (2.8)
Postoperative period,		202.9 (98.5)
days (*n*=105)		(Range, 42–371)

BMI, body mass index; SD, standard deviation.

Data are presented as mean (SD) for continuous variables and as *n* (%) for categorical variables.

### Descriptive statistics of dysfunction after gastrectomy, health literacy, and perceived social support

The mean DAUGS20 total score was 32.8 ​± ​14.9. The Cronbach's alpha of the scale was 0.91 in this study. Comparing the severity of each symptom on the subscales (i.e., the mean score divided by the number of items in parentheses) revealed that symptom severity was greater for dumping syndrome (2.5), limited activity due to decreased food consumption (2.4), and diarrhoea (2.3) than for other items in the scale. The mean FCCHL total score was 2.7 ​± ​0.5, with critical health literacy having the lowest score of 2.3 ​± ​0.7, followed by communicative health literacy at 2.7 ​± ​0.7. The Cronbach's alpha of the scale was 0.81 in this study. The mean MSPSS total score was 5.3 ​± ​1.1, with friend support having the lowest score of 4.3 ​± ​1.6, followed by support by a significant other with a score of 5.7 ​± ​1.2 (Table 2). The Cronbach's alpha of the scale was 0.91 in this study.

**Table 2:** Descriptive statistics of dysfunction after gastrectomy, health literacy, and perceived social support.

	Point range	Mean (mean divided by the number of items)	SD	95% CI
**Dysfunction after gastrectomy (DAUGS20)**				
Total	0–100	32.8	14.9	[30.3, 35.2]
Symptoms of dumping syndrome	0–15	7.4 (2.5)	3.3	[6.9, 8.0]
Limited activity due to decreased food consumption	0–15	7.2 (2.4)	3.2	[6.6, 7.7]
Diarrhoea symptoms	0–10	4.5 (2.3)	2.7	[4.0, 4.9]
Deglutition dysfunction	0–20	4.1 (2.1)	3.4	[3.5, 4.6]
Hypoglycaemic symptoms	010	2.7 (1.4)	2.1	[2.4, 3.1]
Transfer dysfunction	0–10	2.8 (1.4)	2.1	[2.5, 3.2]
Gastroesophageal reflux	0–20	4.1 (1.0)	3.6	[3.5, 4.7]
**Health literacy (FCCHL)**				
Total	1–4	2.7	0.5	[2.6, 2.7]
Functional health literacy	1–4	2.9	0.7	[2.8, 3.0]
Communicative health literacy	1–4	2.7	0.7	[2.5, 2.8]
Critical health literacy	1–4	2.3	0.7	[2.2, 2.5]
**Perceived social support (MSPSS)**				
Total	1–7	5.3	1.1	[5.1, 5.4]
Family support	1–7	5.8	1.3	[5.6, 5.6]
Support by a significant other	1–7	5.7	1.2	[5.5, 5.9]
Friend support	1–7	4.3	1.6	[4.4, 4.6]

CI, confidence interval; DAUGS20, Dysfunction After Upper Gastrointestinal Surgery 20 scoring system; FCCHL, Functional Communicative and Critical Health Literacy scale; MSPSS, Multidimensional Scale of Perceived Social Support; SD, standard deviation.

### Descriptive statistics and distribution of coping flexibility

**Fig. 2:**
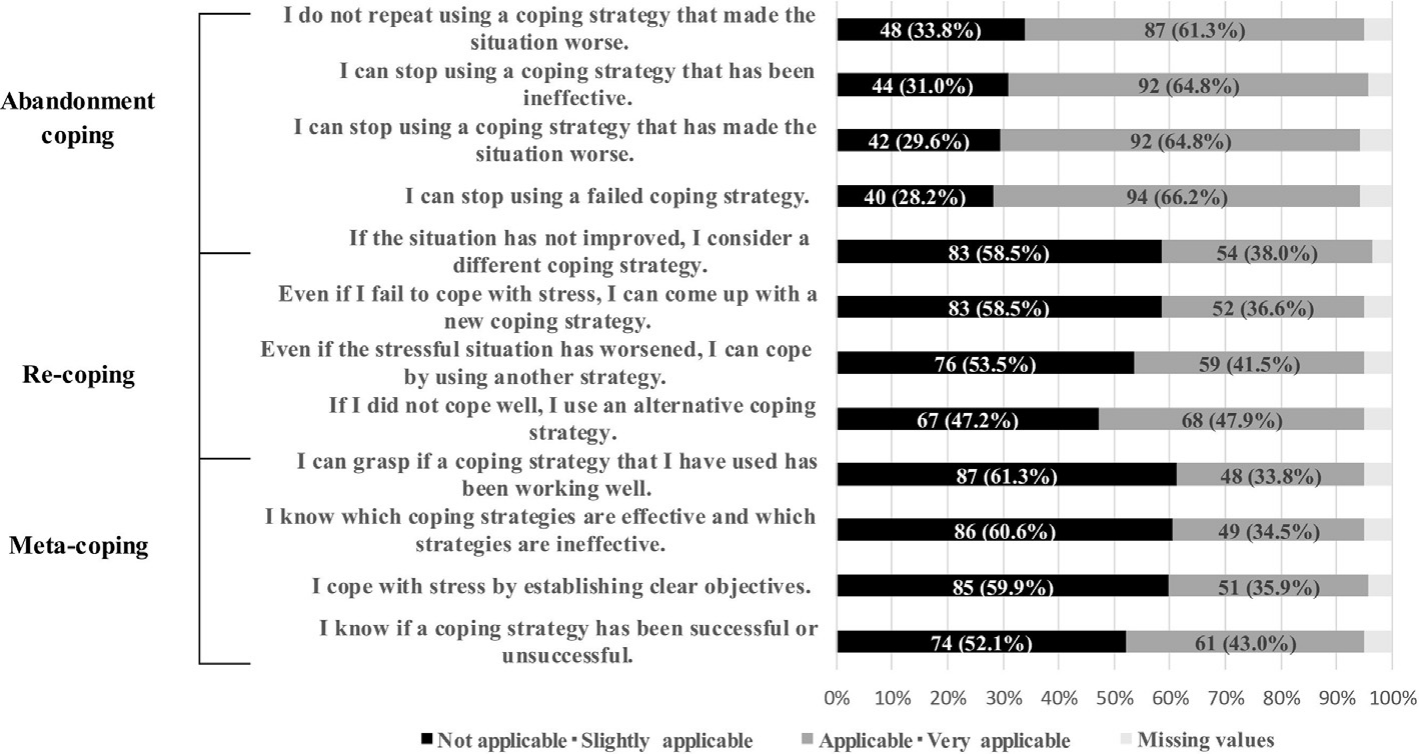
Distribution of items in the Coping Flexibility Scale-Revised.

The mean CFSR total score was 16.2 ​± ​8.9. The Cronbach's alpha of the scale was 0.95 in this study. The mean score for Meta-Coping was 4.8 ​± ​3.0, and items with the highest proportion of ‘not applicable’ or ‘slightly applicable’ responses were ‘I can grasp if a coping strategy that I have used has been working well’ (61.3%), ‘I know which coping strategies are effective and which strategies are ineffective’ (60.6%), and ‘I cope with stress by establishing clear objectives’ (59.9%). The mean score for Re-Coping was 5.0 ​± ​3.1, and the items with the highest proportion of ‘not applicable’ or ‘slightly applicable’ responses were ‘If the situation has not improved, I consider another coping strategy’ (58.5%) and ‘Even if I fail to cope with stress, I can come up with a new coping strategy’ (58.5%). Abandonment Coping had a mean score of 6.4 ​± ​3.5, with approximately 30% of responses being ‘not applicable’ or ‘slightly applicable’ for all items (Table 3 and Fig. 2).

**Table 3:** Descriptive statistics of coping flexibility.

	Point range	Mean	SD	95% CI
**Total CFSR**	0–36	16.2	8.9	[14.7, 17.7]
**Abandonment coping**	0–12	6.4	3.5	[5.8, 6.9]
**Re-coping**	0–12	5.0	3.1	[4.5, 5.5]
**Meta-coping**	0–12	4.8	3.0	[4.3, 5.3]

CFSR, Coping Flexibility Scale-Revised; CI, confidence interval; SD, standard deviation.

### Determinants of coping flexibility in gastric cancer patients after gastrectomy

Univariable analysis showed that Abandonment Coping was associated with age, income, symptoms of dumping syndrome, limited activity due to decreased food consumption, and communicative health literacy. Re-Coping was associated with symptoms of dumping syndrome, limited activity due to decreased food consumption, and support from significant others and friends. Multivariable analysis showed that higher levels of limited activity due to decreased food consumption were associated with lower scores for Abandonment Coping (OR: 0.4; *P* ​= ​0.03; 95% CI, 0.2–0.9) and Re-Coping (OR: 0.3; *P* ​= ​0.003; 95% CI, 0.1–0.6). Higher communicative health literacy was associated with higher Abandonment Coping scores (OR: 1.1; *P* ​= ​0.04; 95% CI, 1.0–1.3), whereas higher critical health literacy was associated with higher Re-Coping scores (OR: 1.2; *P* ​= ​0.03; 95% CI, 1.0–1.3; Table 4, Table 5). Univariable and multivariable analyses revealed no significant variable for Meta-Coping (Table 6).

**Table 4:** Determinants of Abandonment Coping in gastric cancer patients after gastrectomy.

Variable	Univariable analysis			Multivariable analysis		
	OR	95% CI	*P* value	OR	95% CI	*P* value
**Participants' characteristics**						
Age	1.0	[0.9, 1.0]	0.03			
Sex (male: 1 and female: 0)	1.8	[0.9, 3.9]	0.11			
Cohabitant (yes: 1 and no: 0)	0.7	[0.3, 1.7]	0.43			
Income (≥ 4 million yen: 1 and < 4 million yen: 0)	2.8	[1.2, 6.8]	0.02			
Current cancer therapy (yes: 1 and no: 0)	1.3	[0.6, 2.6]	0.50			
Comorbidity (yes: 1 and no: 0)	1.0	[0.5, 2.0]	0.94			
**Dysfunction after gastrectomy (DAUGS20)**						
Symptoms of dumping syndrome	0.4	[0.2, 0.9]	0.02			
Diarrhoea symptoms	1.7	[0.9, 3.4]	0.13			
Limited activity due to decreased food consumption	0.4	[0.2, 0.9]	0.03	0.4	[0.2, 0.9]	0.03
Hypoglycaemic symptoms	0.7	[0.3, 1.5]	0.35			
Transfer dysfunction	0.7	[0.4, 1.5]	0.37			
Deglutition dysfunction	0.7	[0.3, 1.8]	0.48			
Gastroesophageal reflux	1.0	[0.4, 2.5]	0.94			
**Health literacy (FCCHL)**						
Functional health literacy	1.1	[0.7, 1.7]	0.77			
Communicative health literacy	1.7	[1.0, 2.8]	0.04	1.1	[1.0, 1.3]	0.04
Critical health literacy	1.4	[0.9, 2.2]	0.14			
**Perceived social support (MSPSS)**						
Family support	1.1	[0.8, 1.4]	0.62			
Support by a significant other	1.1	[0.9, 1.5]	0.34			
Friend support	1.2	[0.9, 1.4]	0.16			

CI, confidence interval; DAUGS20, Dysfunction After Upper Gastrointestinal Surgery 20 scoring system; FCCHL, Functional Communicative and Critical Health Literacy scale; MSPSS, Multidimensional Scale of Perceived Social Support; OR, odds ratio.

Abandonment Coping scores were defined as high for the group scoring above the mean and low for the group scoring below the mean.

The *P* values are estimated, based on univariable and multiple logistic regression analysis.

**Table 5:** Determinants of Re-Coping in gastric cancer patients after gastrectomy.

Variable	Univariable analysis			Multivariable analysis		
	OR	95% CI	*P* value	OR	95% CI	*P* value
**Participants' characteristics**						
Age	1.0	[0.9, 1.0]	0.10			
Sex (male: 1 and female: 0)	1.4	[0.6, 2.8]	0.42			
Cohabitant (yes: 1 and no: 0)	0.9	[0.4, 2.2]	0.83			
Income (≥ 4 million yen: 1 and < 4 million yen: 0)	2.2	[1.0, 5.2]	0.07			
Current cancer therapy (yes: 1 and no: 0)	0.7	[0.4, 1.5]	0.39			
Comorbidity (yes: 1 and no: 0)	0.7	[0.3, 1.5]	0.37			
**Dysfunction after gastrectomy (DAUGS20)**						
Symptoms of dumping syndrome	0.5	[0.2, 1.0]	0.05			
Diarrhoea symptoms	1.4	[0.7, 2.8]	0.33			
Limited activity due to decreased food consumption	0.4	[0.2, 0.8]	0.01	0.3	[0.1, 0.6]	0.003
Hypoglycaemic symptoms	0.8	[0.4, 1.7]	0.62			
Transfer dysfunction	0.5	[0.2, 1.0]	0.06			
Deglutition dysfunction	0.5	[0.2, 1.4]	0.18			
Gastroesophageal reflux	1.0	[0.4, 2.4]	0.94			
**Health literacy (FCCHL)**						
Functional health literacy	0.9	[0.5, 1.4]	0.57			
Communicative health literacy	1.5	[0.9, 2.4]	0.11			
Critical health literacy	1.6	[1.0, 2.5]	0.06	1.2	[1.0, 1.3]	0.03
**Perceived social support (MSPSS)**						
Family support	1.3	[0.9, 1.7]	0.13			
Support by a significant other	1.5	[1.1, 2.0]	0.02			
Friend support	1.3	[1.0, 1.6]	0.03			

CI, confidence interval; DAUGS20, Dysfunction After Upper Gastrointestinal Surgery 20 scoring system; FCCHL, Functional Communicative and Critical Health Literacy scale; MSPSS, Multidimensional Scale of Perceived Social Support; OR, odds ratio.

Re-coping scores were defined as high for the group scoring above the mean and low for the group scoring below the mean.

The *P* values are estimated, based on univariable and multiple logistic regression analysis.

**Table 6:** Determinants of Meta-Coping in gastric cancer patients after gastrectomy.

Variable	Univariable analysis			Multivariable analysis^a^		
	OR	95% CI	*P* value	OR	95% CI	*P* value
**Participants' characteristics**						
Age	1.0	[1.0, 1.0]	0.78			
Sex (male: 1 and female: 0)	0.9	[0.4, 1.8]	0.72			
Cohabitant (yes: 1 and no: 0)	0.8	[0.3, 1.9]	0.60			
Income (≥ 4 million yen: 1 and < 4 million yen: 0)	1.7	[0.7, 4.0]	0.20			
Current cancer therapy (yes: 1 and no: 0)	1.0	[0.5, 2.0]	0.99			
Comorbidity (yes: 1 and no: 0)	1.6	[0.8, 3.5]	0.21			
**Dysfunction after gastrectomy (DAUGS20)**						
Symptoms of dumping syndrome	0.8	[0.4, 1.7]	0.59			
Diarrhoea symptoms	1.2	[0.6, 2.3]	0.66			
Limited activity due to decreased food consumption	0.5	[0.3, 1.1]	0.09			
Hypoglycaemic symptoms	1.3	[0.6, 2.7]	0.49			
Transfer dysfunction	1.0	[0.5, 2.1]	0.96			
Deglutition dysfunction	0.8	[0.3, 2.1]	0.66			
Gastroesophageal reflux	1.1	[0.5, 2.7]	0.82			
**Health literacy (FCCHL)**						
Functional health literacy	0.9	[0.5, 1.5]	0.65			
Communicative health literacy	1.4	[0.9, 2.3]	0.15			
Critical health literacy	1.6	[1.0, 2.5]	0.07			
**Perceived social support (MSPSS)**						
Family support	1.1	[0.8, 1.4]	0.62			
Support by a significant other	1.1	[0.8, 1.4]	0.62			
Friend support	1.2	[1.0, 1.5]	0.10			

CI, confidence interval; DAUGS20, Dysfunction After Upper Gastrointestinal Surgery 20 scoring system; FCCHL, Functional Communicative and Critical Health Literacy scale; MSPSS, Multidimensional Scale of Perceived Social Support; OR, odds ratio.

Meta-coping scores were defined as high for the group scoring above the mean and low for the group scoring below the mean.

The *P* values are estimated, based on univariable and multiple logistic regression analysis.

a Multivariable analyses reveal no significant variables.

## Discussion

### Determinants of coping flexibility

To the best of our knowledge, this study is the first to demonstrate a relationship between postgastrectomy dysfunction and coping flexibility in patients with gastric cancer. An important aspect of this study is the identified association between specific symptoms of postgastrectomy dysfunction and coping flexibility. Participants who experienced a significant decrease in activity due to decreased food intake had a lower ability to abandon ineffective coping strategies and to develop and implement alternative coping methods.

The impact of reduced activity due to decreased food intake on the participants’ coping flexibility may be attributed to the diverse, complex, and fluctuating nature of the symptoms that cause reduced food intake. Postoperative small stomach syndrome, resulting from reduced gastric volume, leads to early satiety after small meals. Changes in gastric morphology and impaired digestion and absorption can cause gastrointestinal symptoms such as diarrhoea and dumping syndrome.^35^ These postgastrectomy dysfunctions contribute to decreased food intake, leading to malnutrition and reduced activity.^35^^,^^38^ The results of the present study showed that the participants were affected by reduced activity due to low food intake, corroborating previous findings.^35^^,^^38^^,^^39^ Reduced activity due to decreased food intake affects coping flexibility. Prolonging this situation can increase stress, potentially leading to anxiety and depression.

To facilitate recovery, patients need to learn about eating and activity strategies adapted to the altered gastric morphology and digestive and absorptive functions. However, the variable nature of postoperative digestive and bowel dysfunction complicates assessing coping strategies, abandoning ineffective strategies, and developing new strategies. Moreover, the available information on the individualised management of postgastrectomy dysfunction is insufficient, and shorter hospital stays further limit opportunities for patients to receive nutritional education in line with their postoperative recovery.^39^ Patients may be confused by the difference between their preoperative expectations of physical recovery and reality and may not recover as expected because of the effects of postgastrectomy dysfunction. They also face challenges in rebuilding their lives as the anticipated recovery is impeded by the effects of postgastrectomy dysfunction.^40^^,^^41^ To effectively adapt coping strategies in response to fluctuating digestive and bowel functions in the postoperative period, abandoning ineffective coping strategies and adopting new ones, with nursing support having a critical role, are essential.^27^^,^^42^

Another important aspect of this study is the reported association between coping flexibility and health literacy. Higher communicative health literacy was associated with a greater ability to abandon ineffective coping strategies, whereas higher critical health literacy was linked to an increased re-coping ability. During the coping process, patients facing stressful situations seek appropriate coping behaviours. Gathering information by consulting health care providers and considering specific coping strategies to reorganise their lives are crucial steps in this process.^26^^,^^28^ Patients who have undergone gastrectomy also seek information regarding the management of postoperative complications. However, owing to the variability in digestive absorption and bowel function after surgery, selecting and applying situationally appropriate information can be challenging.^27^

Coping flexibility is essential for managing postoperative dysfunction. Collecting information about postgastrectomy dysfunction and appropriate coping methods, and accurately assessing the effectiveness of current strategies, are beneficial in helping patients abandon ineffective coping strategies.^27^ Furthermore, critically evaluating the available information and assessing its applicability to the situation is important for developing and implementing new coping strategies. This process requires effective communication and support from health care providers, including nurses.

Given the limited research in this area, the present findings suggest that health literacy has an important role in facilitating coping flexibility. To effectively modify coping strategies in response to changing circumstances, patients must evaluate the effectiveness of their current strategies, abandon those that are ineffective, and acquire alternative approaches, based on new information. Nursing support is essential in this process.

However, the present study did not find a significant association between coping flexibility and social support. This finding may be because of differences in individual perceptions and the use of social support. Specific forms of support and information provision can influence coping flexibility. Future research should explore the nature of social support, individual acceptance, and interactions with other factors to examine their relationship with coping flexibility in more detail.

### Implications for nursing practice and research

Nursing support is essential for assisting patients with postgastrectomy dysfunction to evaluate the effectiveness of their coping strategies, abandon ineffective strategies, and develop alternative strategies, based on new information. First, during patient communication, nurses enquire about symptom mechanisms and patterns to gain a detailed understanding of current coping strategies and their consequences. The nurses then assess the effectiveness of the coping strategies with the patient and advise patients to abandon ineffective coping strategies. Information is also provided on specific coping strategies adapted to the patient's lifestyle with the aim of developing and implementing alternative strategies. It is important to provide patients with practical advice on their diet and meal timing. Thus, by improving health literacy through the provision of information tailored to individual situations and supporting the patient's ability to effectively acquire necessary information, it is expected that a patient's ability to select and effectively implement the most appropriate coping strategies in various situations will be improved. Increased coping flexibility may improve the quality of life of gastric cancer patients after gastrectomy.

### Limitations

The following limitations must be considered when interpreting the results of this study. First, because this was a cross-sectional study, causal relationships between variables cannot be inferred.

Second, the study results are geographically and culturally limited to Japan, and the single time-point assessment further limits its applicability to different stages of recovery and patient populations.

Third, including only patients who agreed to participate may have resulted in overrepresentation of those with better outcomes. Additionally, the exclusion of patients with cancer recurrence or serious complications may have biased the results toward patients with better recovery and coping flexibility. Other influential factors, such as psychological interventions or family support systems, may affect participants’ coping flexibility and should be adequately controlled.

Finally, the sample size was relatively small, which may have affected the robustness of the results. The sample size was determined based on a medium effect size, which may limit the generalisability of the results.

To overcome these limitations, longitudinal studies are warranted to examine changes in coping flexibility over time and to develop interventions tailored to different stages of recovery. Such research would provide a more comprehensive understanding of the dynamic relationship between coping flexibility and postgastrectomy dysfunction, thereby facilitating the development of targeted and effective nursing interventions.

## Conclusions

This study investigated the current situation and factors associated with coping flexibility in patients after gastrectomy. Patients with reduced activity due to decreased food intake tended to have a lower ability to abandon ineffective coping strategies and develop and implement new coping strategies, thereby suggesting an association between health literacy and coping flexibility. Nursing support that allows flexible changes in coping strategies in response to fluctuating digestive and bowel functions is important for facilitating postoperative adaptation. Understanding symptoms through communication with the patient, assessment and advice on coping strategies, provision of specific information, and ongoing follow-ups are recommended. Increased coping flexibility may improve the quality of life of gastric cancer patients after gastrectomy. The results of this study provide important suggestions for improving the care of postgastrectomy patients and their quality of life.

## CRediT authorship contribution statement

**Miwako Eto**: Conceptualisation, Methodology, Funding acquisition, Data collection, Data curation, Formal analysis, Writing – original draft. **Sena Yamamoto**: Conceptualisation, Methodology, Formal analysis, Writing – review & editing. **Ryohei Kawabata**: Investigation. **Tamon Miyanaga**: Investigation. **Noriko Iga**: Investigation. **Aoi Yoshino**: Investigation. **Hiroshi Yamada**: Investigation. **Yoko Nishitani**: Investigation. **Mami Matsunaga**: Investigation. **Harue Arao**: Conceptualisation, Formal analysis, Writing – review & editing, Supervision. All authors had full access to all data in the study, and the corresponding author had the final responsibility for the decision to submit the manuscript for publication. The corresponding author attests that all listed authors meet the authorship criteria and that no individual meeting the criteria has been omitted.

## Ethics statement

The study was approved by the Clinical Research Ethics Committees of Bell-land General Hospital (Osaka, Japan; IRB No. 2022-010, 19 August 2022), Fukui Prefectural Hospital (Fukui, Japan; IRB No. 23-24, 22 September 2023), Kita-Harima Medical Center (Hyogo, Japan; IRB No. 05-27, 27 July 2023), Japanese Red Cross Kyoto Daini Hospital (Kyoto, Japan; IRB No. S2022-39, 23 December 2022), Ikeda City Hospital (Osaka, Japan; IRB No. 3462, 28 October 2022), Sakai City Medical Center (Osaka, Japan; IRB No. 22-322, 2 November 2022), and Kishiwada City Hospital (Osaka, Japan; IRB No. 36, 11 October 2022). Fuchu Hospital (Osaka, Japan) approved the study, based on the results of an ethical review by Bell-land General Hospital. All participants provided written informed consent.

## Funding

This study was supported by an SGH Cancer Nursing Research Grant. The funder had no role in considering the study design or in the collection, analysis, interpretation of data, writing of the report, or deciding to submit the article for publication.

## Data availability statement

The data that support the findings of this study are available from the corresponding author, H.A., upon reasonable request.

## Declaration of generative AI and AI-assisted technologies in the writing process

No AI tools/services were used during the preparation of this work.

## Declaration of competing interest

The authors declare no conflict of interest.

## References

[1] World Health Organization (2022). Cancer.

[2] Sharma R. (2024). Burden of stomach cancer incidence, mortality, disability-adjusted life years, and risk factors in 204 countries, 1990–2019: an examination of global burden of disease 2019. J Gastrointest Cancer.

[3] Smyth E.C., Nilsson M., Grabsch H.I., van Grieken N.C., Lordick F. (2020). Gastric cancer. Lancet.

[4] Eom S.S., Choi W., Eom B.W. (2022). A comprehensive and comparative review of global gastric cancer treatment guidelines. J Gastric Cancer.

[5] Samrat R., Naimish M., Samiran N. (2020). Post-gastrectomy complications - an overview. Chirurgia.

[6] Kim C.Y. (2022). Postgastrectomy syndrome. Foregut Surg.

[7] Nakada K. (2016). Postgastrectomy syndrome: up to date. J Jpn Surg Assoc.

[8] Şayır D., Karacabay K. (2024). Nutritional experiences of patients undergoing total gastrectomy surgery: a qualitative study. Gastroenterol Nurs.

[9] Kouhestani M., Ahmadi Gharaei H., Fararouei M., Hosienpour Ghahremanloo H., Ghaiasvand R., Dianatinasab M. (2022). Global and regional geographical prevalence of depression in gastric cancer: a systematic review and meta-analysis. BMJ Support Palliat Care.

[10] Kim G.M., Kim S.J., Song S.K. (2017). Prevalence and prognostic implications of psychological distress in patients with gastric cancer. BMC Cancer.

[11] Kato T. (2012). Development of the Coping Flexibility Scale: evidence for the coping flexibility hypothesis. J Counsel Psychol.

[12] Kato T., Basinska M.A. (2015). Coping Flexibility with Stress in Health and in Disease.

[13] Kato T. (2020). Examination of the coping flexibility hypothesis using the Coping Flexibility Scale-Revised. Front Psychol.

[14] Cheng C., Kogan A., Chio J.H. (2012). The effectiveness of a new, coping flexibility intervention as compared with a cognitive-behavioural intervention in managing work stress. Work Stress.

[15] Rudnik A., Piotrowicz G., Basińska M.A., Rashedi V. (2019). The importance of cognitive flexibility and flexibility in coping with stress for the quality of life in inflammatory bowel disease patients during biological therapy. A preliminary report. Prz Gastroenterol.

[16] Knowles L.M., O'Connor M.F. (2015). Coping flexibility, forward focus and trauma focus in older widows and widowers. Bereave Care.

[17] Basińska M.A., Sołtys M. (2020). Personal resources and flexibility in coping with stress depending on perceived stress in a group of cancer patients. Health Psychol Rep.

[18] Dahabre R., Roziner I., Bentley G. (2022). The moderating role of coping flexibility in reports of somatic symptoms among early breast cancer patients. Soc Sci Med.

[19] Nakamura S. (2015). Effects of an intervention program for improving coping flexibility. Bulletin of the Graduate School of Education, Hiroshima University. Education and Human Science.

[20] Zimmer-Gembeck M.J. (2021). Coping flexibility: variability, fit and associations with efficacy, emotion regulation, decentering and responses to stress. Stress Health.

[21] Vriezekolk J.E., van Lankveld W.G.J.M., Eijsbouts A.M.M., van Helmond T., Geenen R., van den Ende C.H.M. (2012). The coping flexibility questionnaire: development and initial validation in patients with chronic rheumatic diseases. Rheumatol Int.

[22] Cheng C., Yang F.C., Jun S., Hutton J.M. (2007). Flexible coping psychotherapy for functional dyspeptic patients: a randomized, controlled trial. Psychosom Med.

[23] Kruczek A., Basińska M.A., Janicka M. (2020). Cognitive flexibility and flexibility in coping in nurses - the moderating role of age, seniority and the sense of stress. Int J Occup Med Environ Health.

[24] Zaken M.D., Boyraz G., Dickerson S.S. (2022). COVID-19 pandemic-related stressors and posttraumatic stress: the main, moderating, indirect, and mediating effects of social support. Stress Health.

[25] Vosbergen S., Peek N., Mulder-Wiggers J.M.R. (2014). An online survey to study the relationship between patients' health literacy and coping style and their preferences for self-management-related information. Patient Prefer Adherence.

[26] Jung M., Ramanadhan S., Viswanath K. (2013). Effect of information seeking and avoidance behavior on self-rated health status among cancer survivors. Patient Educ Counsel.

[27] Eto M., Yamamoto S., Arao H. (2022). Flexible coping in cancer care: a concept analysis. Cancer Care Res Online.

[28] Lazarus R.S., Folkman S. (1984).

[29] Faul F., Erdfelder E., Lang A.G., Buchner A. (2007). G∗Power 3: a flexible statistical power analysis program for the social, behavioral, and biomedical sciences. Behav Res Methods.

[30] von Elm E., Altman D.G., Egger M., Pocock S.J., Gøtzsche P.C., Vandenbroucke J.P. (2007). The strengthening the reporting of observational studies in Epidemiology (STROBE) statement: guidelines for reporting observational studies. PLoS Med.

[31] Nakamura M., Kido Y., Yano M., Hosoya Y. (2005). Reliability and validity of a new scale to assess postoperative dysfunction after resection of upper gastrointestinal carcinoma. Surg Today.

[32] Nakamura M., Kido Y., Hosoya Y., Yano M., Nagai H., Monden M. (2007). Postoperative gastrointestinal dysfunction after 2-field versus 3-field lymph node dissection in patients with esophageal cancer. Surg Today.

[33] Nakamura M., Kido Y., Egawa T. (2008). Development of a 32-item scale to assess postoperative dysfunction after upper gastrointestinal cancer resection. J Clin Nurs.

[34] Nakamura M., Hosoya Y., Yano M. (2011). Extent of gastric resection impacts patient quality of life: the Dysfunction after Upper Gastrointestinal Surgery for Cancer (DAUGS32) scoring system. Ann Surg Oncol.

[35] Nakamura M., Hosoya Y., Umeshita K. (2011). Postoperative quality of life: development and validation of the ‘Dysfunction after Upper Gastrointestinal Surgery’ scoring system. J Am Coll Surg.

[36] Ishikawa H., Takeuchi T., Yano E. (2008). Measuring functional, communicative, and critical health literacy among diabetic patients. Diabetes Care.

[37] Iwasa H., Gondo Y., Masui Y. (2007). Reliability and validity of the Japanese version of the multidimensional scale of perceived social support: a study on middle-aged and older adults. J Health Welf Stat.

[38] Gharagozlian S., Mala T., Brekke H.K., Kolbjørnsen L.C., Ullerud Å.A., Johnson E. (2020). Nutritional status, sarcopenia, gastrointestinal symptoms and quality of life after gastrectomy for cancer–A cross-sectional pilot study. Clin Nutr ESPEN..

[39] Noguchi A., Sato F., Sasaki K. (2023). Relationship between physical symptoms and self-care ability in postoperative gastric cancer. Bull Fukushima Med Univ Sch Nurs.

[40] Kosaka M., Majima T. (2011). Structure of flexible coping for postoperative gastric cancer patients receiving ambulatory chemotherapy. J Chiba Acad Nurs Sci..

[41] Yamawaki K., Fujita M. (2006). Stress and coping associated with resumption of work in people after surgery for stomach cancer. J Jpn Soc Cancer Nurs..

[42] Jennifer P.O., Juyeon P. (2016). Coping with the interpersonal stresses of bariatric surgery: an interpretive study of women's experiences. Int J Psychol Counsel.

